# Efficient eco-friendly biochar-based catalyst design for CO_2_ conversion via the reverse water-gas shift reaction

**DOI:** 10.3389/fchem.2026.1953213

**Published:** 2026-10-27

**Authors:** Maryanne Mosonik, Qi Zhang, Juan J. Villora-Pico, Tomas Ramirez Reina, Roberto Volpe

**Affiliations:** 1 School of Engineering and Materials Science, Queen Mary University of London, London, United Kingdom; 2 Department of Chemical and Process Engineering, University of Surrey, Guildford, United Kingdom; 3 GreenX Technology Ltd., Thame, Oxfordshire, United Kingdom; 4 Inorganic Chemistry Department and Materials Sciences Institute, University of Seville-CSIC, Seville, Spain

**Keywords:** biochar-based catalyst, CO-selective catalysis, iron catalyst, reverse water-gas shift reaction, sabatier reaction suppression

## Abstract

**Introduction:**

Eco-friendly catalysts for CO_2_ conversion reactions are gathering momentum within the ongoing transition to a low-carbon economy. Here, we present a new family of biochar-based Fe catalysts for high temperature reverse water-gas shift (RWGS) reaction.

**Methods:**

Almond-shell-derived biochar was prepared by pyrolysis under an argon atmosphere at 500 °C. Fe-based catalysts with iron loadings of 20 and 40 wt% were synthesised by wet impregnation, followed by pyrolysis at 700 or 1000 °C for different holding times.

**Results:**

At 750 °C, the synthesized catalysts exhibited CO_2_ conversions ranging from 74% to 87% and CO selectivities between 82% and 100%, with four of the five catalysts achieving conversions above 80% and approximately 100% CO selectivity. Importantly, no CH_4_ was detected during the RWGS reaction under the tested conditions, despite the high H_2_/CO_2_ ratio of 4:1.

**Discussion:**

Our biochar based systems display an complex interplay of active phases Fe^0^, Fe_3_C and Fe_3_O_4_, with metallic iron being the dominant active component responsible for commendable catalytic performance.

## 1 Introduction

The rising emissions of carbon dioxide (CO_2_) have been a universal cause of concern, as its adverse environmental effects may become irreversible should the current emissions trajectory persist. Carbon capture and storage (CCS) and carbon capture and utilisation (CCU) are among the solutions researchers and governments are proposing to reduce the amount of CO_2_ in the atmosphere (Zhu et al., 2020). RWGS reaction, is an example of CCU that is used to convert CO_2_ into CO (González-Castaño et al., 2021).

RWGS can be coupled to a Fischer - Tropsch synthesis (FTS), a chemical process that converts syngas into liquid hydrocarbon fuels. Besides integrated schemes comprising RWGS-Methanol synthesis units (CAMERE process) are envisaged as direct routes for CO_2_ conversion to added value products (Joo et al., 1999; Chou et al., 2019). Hence, advanced catalysts design and optimisation for the RWGS reaction constitute a current challenge within the catalysis and green chemistry communities. In particular, emphasis has been on monometallic catalysts such as Rh (Boffa et al., 1994), Pd (Kwak et al., 2013), Cu (Chen et al., 2001), Ni (Wang et al., 2008), Fe (Loiland et al., 2016), bimetallic catalysts such as Fe-Mo (Kharaji et al., 2013) and transition metal carbide catalysts (Porosoff et al., 2014; Posada-Pérez et al., 2014). Catalysts’ supports, such as inert Al_2_O_3_ (Loiland et al., 2016) or reducible oxides such as CeO_2_ (Porosoff and Chen, 2013), have been studied in particular with respect to their influence on CO/CH_4_ selectivity (Kwak et al., 2013; Zhao et al., 2021). More recently, CeO_2_-based catalysts incorporating Fe, Ru and oxygen vacancies have also been investigated for enhanced RWGS activity (Jiang et al., 2025). Extensive research has been published on Fe as catalysts, but studies on the use of biochar as support and the influence of Fe/biochar interaction on RWGS are still lacking. Yet, porous carbon materials such as biochars are desirable supports as their textural/morphological properties and their surface chemistry can be tuned by thermal and/or chemical treatments to enhance their performance (Figueiredo et al., 1999; Prati and Rossi, 1998; Demirel et al., 2007). Biochar, a product of the thermochemical decomposition of biomass in an inert atmosphere (pyrolysis), has been proposed for catalysis owing to their highly porous and carbon rich nature, however, only a few of these studies have focused on RWGS reaction (Lee et al., 2017). For instance, Biochar-based Pt catalysts were proposed by Santos et al. to evaluate their effect on WGS and RWGS and the promotional effect of Na (Santos et al., 2018). The authors found that Na enhanced catalytic performance and CO methanation was favoured. Recent studies have also explored CO_2_ methanation under photothermal conditions (Jiang et al., 2023). Indeed decoupling methanation and RWGS and delivering fully CO selective catalysts is a major progress for real-world RWGS applications. In this study, we demonstrated that eco-friendly biochar-based Fe catalysts can overcome CO_2_ methanation favouring CO selectivity in a broad temperature window offering flexible scenarios for the RWGS.

## 2 Materials and methods

### 2.1 Catalysts preparation

Almond Shells (AS) sourced from Southern Italy were milled and sieved to particle size lower than 125 μm, oven dried for 48 h at 105 °C, and stored at −20 °C in Argon atmosphere to prevent aging and moisture intake during storage. Dried AS was pyrolysed in single 5g batches, in 10 mL alumina crucibles covered by an alumina lid, in a Lenton® 12/50/100 series tubular furnace for 1 h at 500 °C in a 1 L min^-1^ Ar flow. Each batch yielded approximately 30% w/w dry basis of biochar sample that was then washed with 20 mL methanol via magnetic stirring, at 40 °C for 24 h. Washed biochar was recovered *via* vacuum filtration and then oven dried at 70 °C for 24 h to eliminate any methanol or moisture intake. Catalysts were prepared by wet impregnation. Fe (20% and 40% w/w) iron (ii) acetate (Fe(CO_2_CH_3_)_2_, was sourced at Aldrich, 95%, and dissolved in 20 mL methanol. Methanol-washed and 1.5 g dried biochar particles were added to this solution and the mixture stirred for 24 h at room temperature. The Fe-impregnated biochar was then recovered by vacuum filtration and then and then oven dried for 24 h at 70 °C. Dried impregnated biochar was then pyrolysed at 700 °C (Low Heat - LH) or 1000 °C (High Heat - HH) for 3 h (Long - L) or 1 h (Short - S) holding temperatures.

### 2.2 Catalysts characterization

The crystalline structures of the samples were characterized by X-Ray Diffraction (XRD) using an X’Pert Pro PANalytical at room temperature using Cu-K^。^(40 mA, 45 kV) over a 10°-90° 2θ range. X’PertHighscore Plus was used to calculate the crystal sizes.

The N_2_ adsorption studies at −196 °C equipped with an Autosorb-6 element (Quantachrome Instruments) were conducted to determine the Brunauer–Emmett–Teller (BET) surface areas. Samples were prepared by out-gassing at 250 °C for 4 h under vacuum. The Barret-Joyner-Halenda (BJH) method was used to estimate the average pore size and pore volume.

The high resolution images were acquired using the Cryo - Transmission Electron Microscope (TEM), model - JEM - F200, using an accelerating voltage of 200 kV.

The Fe content was determined by inductively couple plasma optical emission spectroscopy (ICP-OES) using an iCAP 7200 ICP-OES Duo spectrometer (from ThermoFisher Scientific). Prior to analysis, the samples were subjected to microwave-assisted digestion using an ETHOS EASY microwave digestion system from Milestone.

### 2.3 Catalytic behaviour

The RWGS reactions were carried out in a vertical continuous fixed bed reactor while reactants and products at atmospheric pressure were recorded in *in-operando, via* an ABB AO2020 Advanced optima process gas analyser. The reactor consisted in a 7 mm inner diameter quartz tube in which 0.25 g of dry catalyst was placed on a quartz wool bed placed in the middle section of the reactor. The sample was heated in flowing N_2_ from room temperature to 350 °C. Then, the N_2_ was replaced by the feed gas mixture of H_2_:CO_2_ = 4:1 at a constant weight-hourly space velocity (WHSV) of 12000 mL g^−1^ h^−1^ and the products of the reaction were evaluated from 350 °C to 750 °C. At each temperature, the gas products were analysed after 20 min of steady-state reaction.

CO_2_ conversion (Equation 1), CO selectivity (Equation 2) and CH_4_ selectivity (Equation 3) could be measured based on the above tests. In addition, the CO_2_ consumption rate (Equation 4) and CO formation rate (Equation 5) were determined and normalized by the mass of the active phase in each catalyst determined by ICP-OES. The relative experimental error in CO_2_ conversion and CO/CH_4_ selectivity in this work was given within ±0.5% (Liu et al., 2019). Here, $n_{CO_{2}in}$ is the initial molar flow (kmol min^-1^) of CO_2_ in the reactant mixture and ${\;n}_{COout},n_{CH_{4}out},n_{CO_{2}out}$ are the outlet molar flows in the product stream of CO, CH_4_ and CO_2_ respectively. Furthermore, $m_{{CO}_{2}in}$ , $m_{{CO}_{2}out}$ and $m_{COout}$ denote the corresponding mass flow rates, where $m_{active\;plase}$ represents the mass of active phase present in the catalyst.

$$CO_{2}\;conversion\;\left(\%\right)=\frac{n_{CO_{2}in}-n_{CO_{2}out}}{n_{CO_{2}in}}\cdot 100$$ (1)

$$CO\;selectivity\;\left(\%\right)=\frac{n_{COout}}{n_{CO_{2}in}-n_{CO_{2}out}}\cdot 100$$ (2)

$${CH}_{4}\;selectivity\;\left(\%\right)=\frac{n_{CH_{4}out}}{n_{CO_{2}in}-n_{CO_{2}out}}\cdot 100$$ (3)

$${CO}_{2}\;consumption\;rate\;\left(g_{{CO}_{2}}\min^{-1}g_{active\;phase}^{-1}\right)=\frac{m_{{CO}_{2}in}-m_{{CO}_{2}out}}{m_{active\;plase}}$$ (4)

$$CO\;production\;rate\;\left(g_{CO}\min^{-1}g_{active\;phase}^{-1}\right)=\frac{m_{COout}}{m_{active\;plase}}$$ (5)

### 2.4 Thermodynamic simulation

ChemStations’ ChemCad software package has been used to observe the thermodynamic limits of the studied reactions over a range of temperatures. The equation of state package used is that of Soave-Redlich-Kwong, with the unit operation being a Gibbs reactor. Material flows into the reactor are identical to those intended to be used for experimentation.

## 3 Results and discussions

### 3.1 Characterization

#### 3.1.1 XRD analysis

Figure 1 displays the XRD pattern of the as synthesised iron catalysts. All the samples are mainly composed of centred cubic α-Fe as identified from the Joint Committee on Powder Diffraction Standards database (JCPDS 65–4899), the corresponding peaks are labelled by red dots (Batista et al., 2015; Pastor-Pérez et al., 2017). The peak located at 26.3^o^ should be assigned to typical (002) facets of graphite carbon (JCPDS 41-1487) (Liu et al., 2016; Jurkiewicz et al., 2018). The peaks labelled by blue dots should be aligned on Fe_3_C (JCPDS 35-0772) (Zhang et al., 2022). For 20% LHS samples, the diffraction peak at 36.3^o^ and 42.1^o^ corresponds to Fe_3_O_4_ (JCPDS 88-0315) (Liu et al., 2019). No Fe_3_O_4_ peaks were detected in the other samples.

**FIGURE 1:**
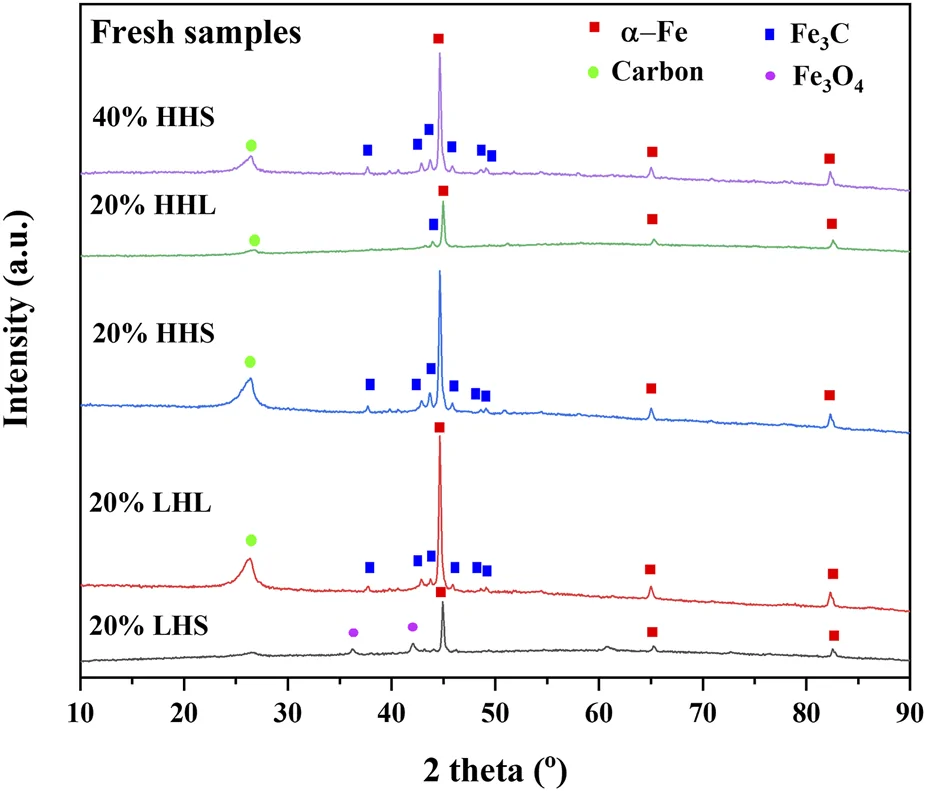
X-ray diffraction patterns for fresh samples.

As can be seen from Figure 1, metallic Fe is the main component for all the studied samples. The Fe_3_C phase appeared at high temperature or longer residence time synthesized catalysts, except the 20% HHL sample. Only one small peak assigned to Fe_3_C phase was detected, in addition, the carbon peak in 20% HHL sample is also smaller than in others. This is assumed to be related to the Fe_3_C decomposition happened under the longer residence time and higher temperature treatment. In previous work by Miki and Ishii (Miki and Ishii, 2014), after heating Fe_3_C under Ar atmosphere, Fe_3_C started to decompose from 527 °C and Fe was observed after 1 hour residence time (Equation 6).

$${Fe}_{3}C\to 3Fe+C$$ (6)

For this work all the catalysts precursors were heated for a minimum of 1 h at temperatures higher or equal to 700 ^o^C, therefore, it is not surprising that Fe^0^ is the main component for all synthesized samples.

The presence of Fe_3_O_4_ in the 20% LHS suggests that reduction of iron (ii) acetate to Fe^0^ and Fe_3_C was incomplete. Iron oxide reduction occurs in three phases; hematite (α-Fe_2_O_3_)/magnetite (Fe_3_O_4_), magnetite (Fe_3_O_4_)/wustite (FeO) and wustite (FeO)/iron (α-Fe) (Kock et al., 1985). FeO only exists in a metastable form below 600 °C, which is why it is not captured by XRD for both holding times at 700 °C (Ali et al., 2011). In the case of biochar supports, it has been widely suggested that iron oxide reduction to iron is aided by gaseous intermediates (CO and H_2_) as opposed to by solid carbon (Fruehan, 1977). CO and H_2_ are produced during pyrolysis in an inert atmosphere.

#### 3.1.2 TEM analysis

A carbon dissolution-precipitation model has been proposed to explain the formation of α-Fe and Fe_3_C species (Neeli and Ramsurn, 2018). In this model, at 1000 °C, carbon atoms dissolve into gaps between the Fe atoms of the face centred cubic, γ-Fe and precipitates out once temperature starts dropping, inadvertently starting the encapsulation process. The encapsulation results in a core-shell structure of iron surrounded by carbon layers as observed in Figure 2.

**FIGURE 2:**
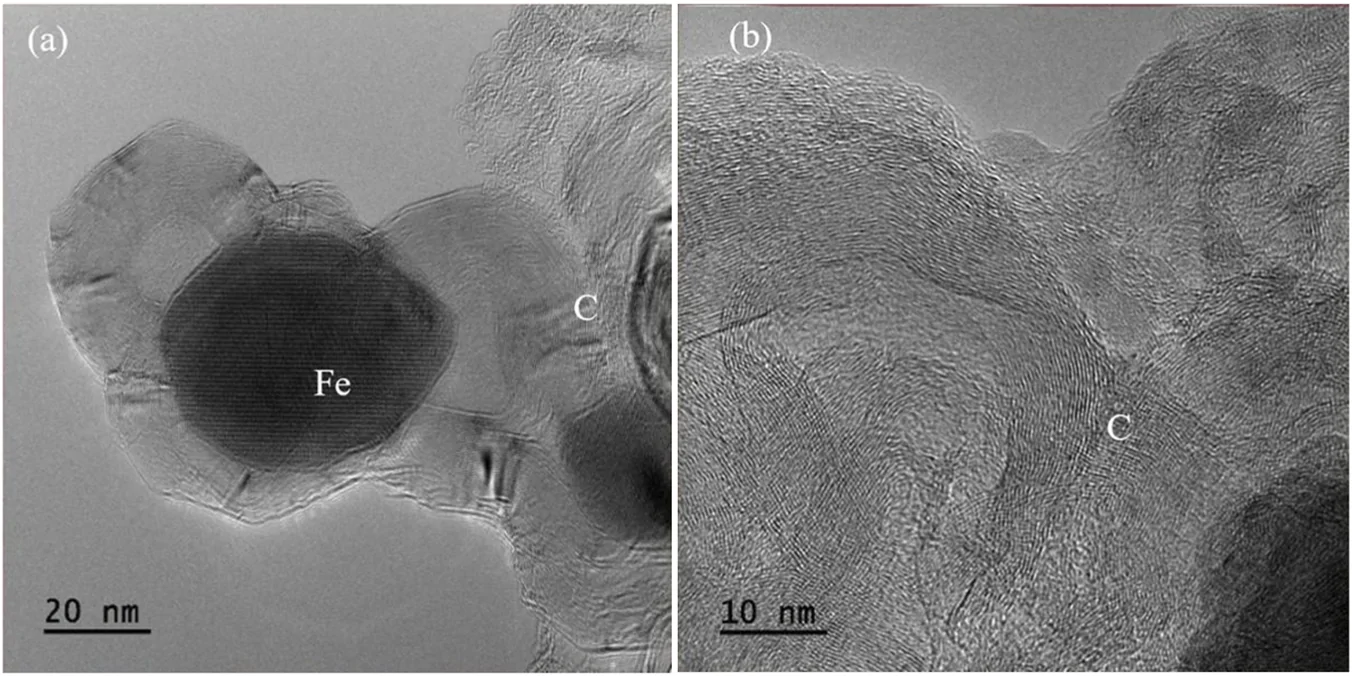
TEM images at different magnifications of the 40% HHS catalyst before the RWGS reaction, showing Fe encapsulated by carbon: **(a)** lower magnification image; **(b)** higher magnification image.

It was found that there was a solubility limit of carbon in iron and that carbon content less than 1.4 wt% resulted in the presence of γ-Fe phase only in the core; higher than 6.7 wt% resulted in full conversion to Fe_3_C and content between 6.7–0.76 wt% resulted in a mixture of both phases (Ding et al., 2006). This diffusion process is slow at 1000 °C, which would suggest that either this process was not complete or the availability of carbon was low resulting in the appearance of both Fe^0^ and Fe_3_C, with a preference to Fe^0^ according to XRD above (Neeli and Ramsurn, 2018). A lower loading of iron on the support would result in disappearance or reduction of the active phase.

#### 3.1.3 Textural properties analysis

The N_2_ adsorption-desorption isotherms for all the fresh samples are shown in Figure 3. The BET surface area, pore volume and pore size of the studied samples are summarized in Table 1. All the surface area value of as synthesized catalysts are during the range from 120 to 150 m^2^/g. The pore sizes of different samples are identical, the pore volume values are also very similar, indicating that the different synthesis conditions only have very limited influence on the textural properties of studied samples. In this case, it is not the determining factor in the RWGS reaction.

**FIGURE 3:**
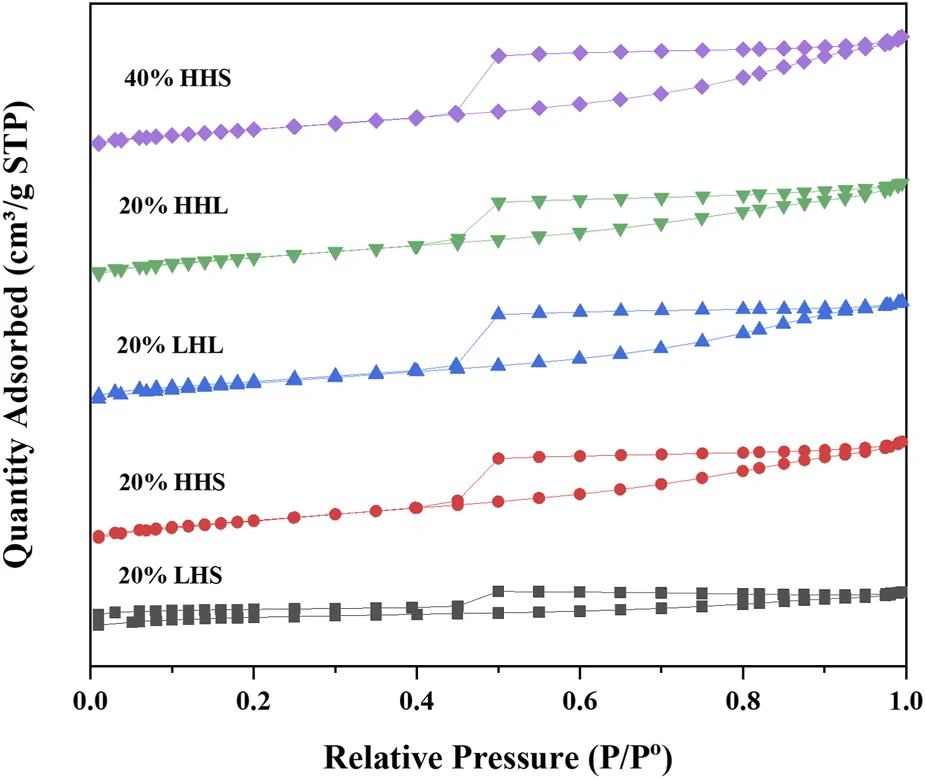
N_2_ adsorption-desorption of all fresh catalysts.

**TABLE 1:** Textural properties of studied samples.

Catalysts	Surface area^a^ (m^2^/g)	Pore volume^b^ (cm^3^/g)	Pore size^c^ (nm)
20% LHS	122	0.08	4.2
20% HHS	140	0.15	4.1
20% LHL	147	0.13	4.0
20% HHL	134	0.14	4.2
40% HHS	124	0.17	4.1

^a^ Calculated by the BET equation.

^b^ Pore volumes calculated from the N2 desorption at a relative pressure of 0.95.

^c^ BJH desorption average pore diameter.

#### 3.1.4 ICP-OES analysis

The actual Fe content of the synthesized catalysts was determined by ICP-OES. The experimentally determined Fe contents were 19.5 wt% and 38.9 wt% for the catalysts with nominal Fe loadings of 20 and 40 wt% respectively.

### 3.2 Catalytic performance

Figure 4a depicts the CO_2_ conversion trends over the prepared catalysts as a function of temperature. The CO selectivities are displayed in Figure 4b. All the synthesized catalysts are active for RWGS in the whole testing temperature range 350–750 °C. As the most common by-products, methane was also measured by our online gas analyser. Despite the high H_2_/CO_2_ ratio used, no methane was detected throughout the investigated temperature range of 350 °C–750 °C, indicating that detectable Sabatier methanation was suppressed under the tested conditions.

**FIGURE 4:**
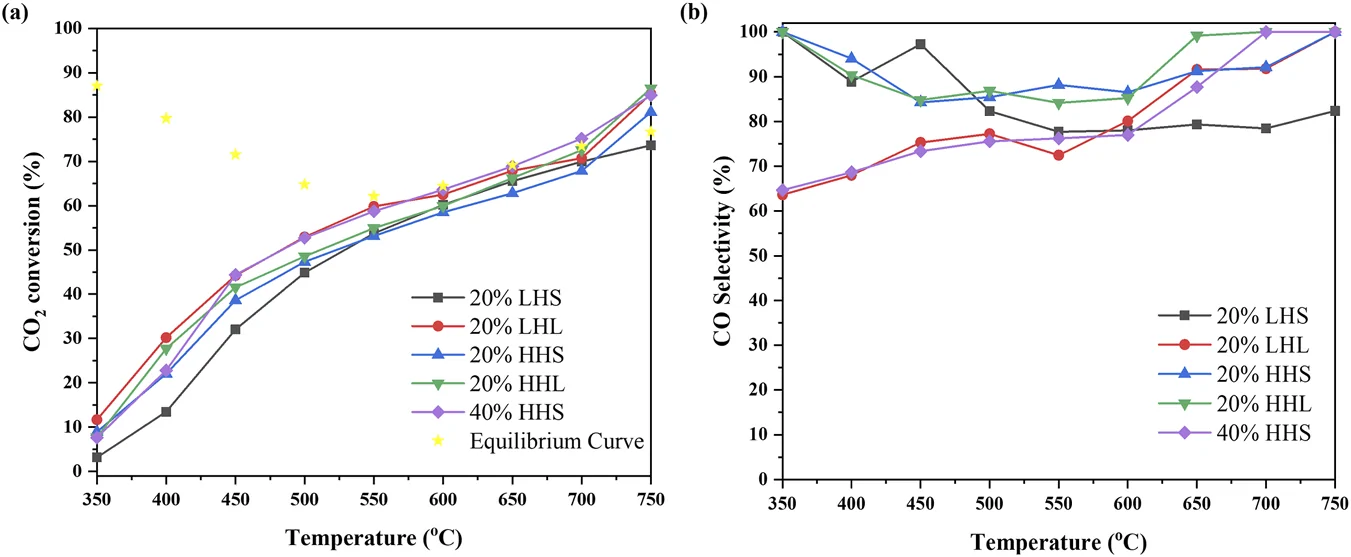
**(a)** CO_2_ conversion **(b)** CO selectivity for studied samples. Reaction conditions: H_2_: CO_2_ = 4:1, WHSV = 12,000 mL g^−1^ h^−1^, T: 350–750 ^o^C, ambient pressure.

In terms of the CO_2_ conversion, 20% LHL and 40% HHS samples exhibited almost identical performance, approaching the calculated equilibrium conversion in the high temperature range (550–750 °C). The CO_2_ conversion towards 20% HHL and 20% HHS samples are slightly lower than the above two. For the 20% LHS sample, the performance value is lower than the rest from 350–500°, but reached at the same level with the temperature increased to 550 °C. The equilibrium CO_2_ conversion shown in Figure 4a was calculated under the experimental conditions of H_2_/CO_2_ = 4:1 and ambient pressure, considering both the RWGS and CO_2_ methanation reactions. Some experimental values slightly exceed the calculated equilibrium line, which may be associated with additional reactions not included in the equilibrium calculation. In particular, biochar gasification may occur at elevated temperatures and contribute to additional CO_2_ consumption and CO formation. In general, the excellent CO_2_ activity is linked to metallic Fe being the main component, which has been previously tested as an active phase for RWGS reaction (Kim et al., 2015; Zhang et al., 2022).

To provide a more meaningful comparison of the catalytic performance, the CO_2_ consumption rates were additionally normalized by the actual mass of active phase in each catalyst (Figure 5a). As expected, the CO_2_ consumption rate increased progressively with reaction temperature for all the catalysts. At lower temperatures (350 °C–450 °C), more important differences were observed among the catalysts, with 20% LHL showing the highest consumption rate at 350 °C and 400 °C, while 20% LHL and 40% HHS exhibited comparable and the highest CO_2_ consumption value at 450 °C. In contrast, 20% LHS displayed the lowest CO_2_ consumption rate within this temperature range. As the reaction temperature increased, the differences among the catalysts became smaller. At 750 °C, 20% LHL, 20% HHL and 40% HHS exhibited the highest rates, reaching approximately 0.32 g CO_2_ g^-1^ active phase min^-1^. Importantly, differences in catalytic activity remained after normalization by the active phase content, indicating that the observed catalytic performance cannot be attributed only to differences in the amount of active phase among the catalysts.

**FIGURE 5:**
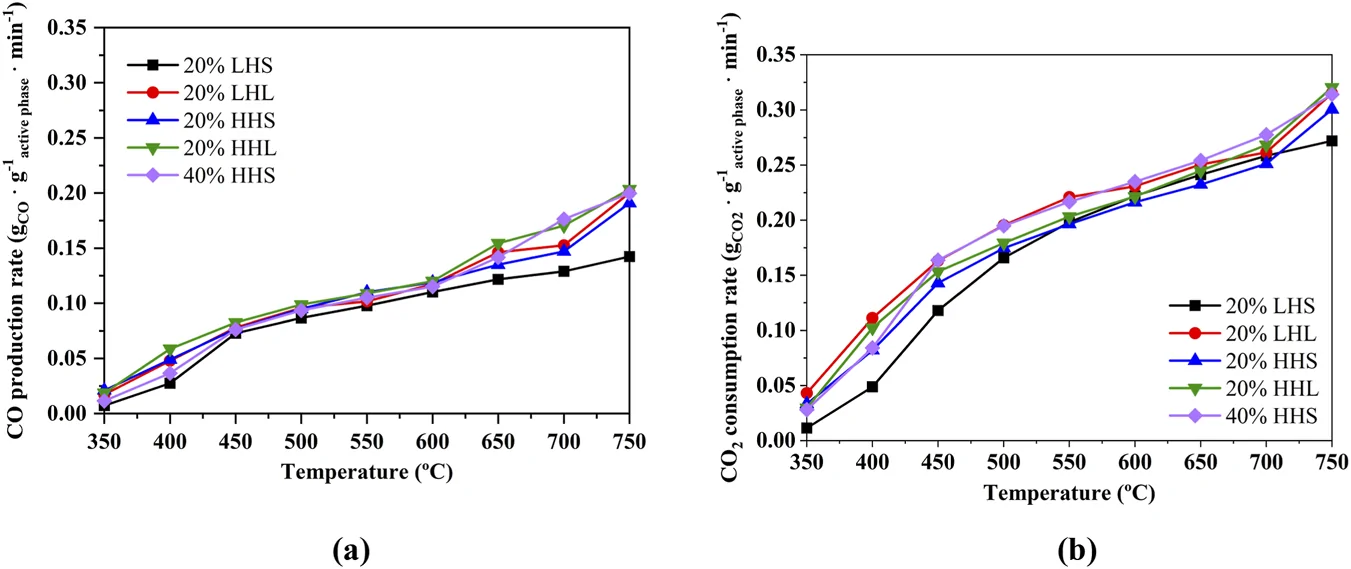
**(a)** CO_2_ consumption rate **(b)** CO formation rate normalized by the mass of active phase for studied catalysts. Reaction conditions: H_2_: CO_2_ = 4:1, WHSV = 12,000 mL g^−1^ h^−1^, T: 350–750 ^o^C, ambient pressure.

A similar trend was observed for the CO formation rate (Figure 5b), which progressively increased with reaction temperature for all catalysts. The differences among the samples were higher at low temperatures and decreased as the reaction temperature increased. At 750 °C, 20% LHL, 20% HHL and 40% HHS exhibited the highest formation rates, whereas 20% HHS showed a slightly lower value. Overall, the similar trends observed for the CO_2_ consumption and CO formation rates are consistent with the high CO selectivity exhibited by the catalysts.

The role of supports in RWGS has been studied in relation to its effect on selectivity and activity. Kwak et al. (2013) demonstrated this by dispersing Pd on Al_2_O_3_ and multiwall carbon nanotubes (MWCNT). The Pd/MWCNT system failed to reduce CO_2_, while the 0.5% Pd dispersed on the inert Al_2_O_3_ reduced CO_2_ at high rates. The dissociation of CO_2_ occurs at the metal/support interface. Then, whether the hydrogenation of CO proceeds or not, depends on the binding energy between the CO and the catalyst (Kattel et al., 2016), whereby the synergy between the metal and support/promoter is proven important as well as the importance bi-functionality in catalysts altogether. Although our present work does not aim to investigate the CO_2_ dissociation mechanism, it is likely that CO binds weakly to the Fe/biochar interface, similar to that described by (Kattel et al., 2016), which may contribute to the absence of detectable CH_4_ under the tested conditions.

Indeed, the mechanism of CO and CH_4_ formation is controversial with two main competing theories discussed in literature. The more widely accepted mechanism proposes the formation of adsorbed CO intermediate before methanation, another theory presented by Medsforth (Samuel Medsforth, 1923) proposed a reaction yielding methane taking place in the gas phase, without the formation of an intermediate (Fisher and Bell, 1996). Park and co-authors support the former theory by stating that an adsorbed CO intermediate is needed before hydrogenation to methane can occur (Park and McFarland, 2009). Their Pd-Mg/SiO_2_ catalyst is shown to stabilise the CO intermediate thereby showing higher selectivity compared to Pd/SiO_2._ According to this finding, when the intermediate is not stabilised, it desorbs, yielding CO. In our case, the lack of inhibition of CO desorption, together with the high CO selectivity observed over Fe, may favour the desorption of CO before further conversion to methane under the tested conditions.

It should be noted that the CO selectivity was below 100% at some temperatures, suggesting that a fraction of the converted CO_2_ was not quantified as CO or CH_4_. Although CH_4_ was not detected by the online gas analyser, the formation of other carbon-containing products cannot be excluded. For example, CO generated through the RWGS reaction could potentially undergo further hydrogenation through Fischer-Tropsch pathways, leading to C_2_^+^ hydrocarbons or oxygenated products. Similar carbon-containing products were identified by mass spectrometric analysis in our previous study of Fe-based RWGS catalysts (Zhang et al., 2022). However, these species were not identified or quantified in the present work. Therefore, the nature and amount of the unaccounted carbon fraction remain undetermined, and a completed carbon balances could not be established.

Finally, the catalytic performance of the catalysts developed in this work was compared with representative Fe-based catalysts reported in literature, as summarized in Table 2. The catalysts exhibited competitive CO_2_ conversion and high CO selectivity under similar conditions compared with other Fe-based catalytic systems. Furthermore, similar CO_2_ consumption rates were observed.

**TABLE 2:** Catalyst performance comparison with materials reported in the literature.

Catalyst	Temperature (°C)	H_2_:CO_2_ ratio	CO_2_ conversion (%)	CO selectivity (%)	CO_2_ conversion rate (g_CO2_ g^-1^_active phase_ min^-1^)	WHSV (ml/g_cat_h)	Ref.
20Fe/CeO_2_	500	4	50	100	0.450	60,000	Gou et al. (2024)
15Fe/CeO_2_-Al_2_O_3_	750	4	75	100	0.833	30,000	Yang et al. (2020)
5Cu5Fe/Sm_0.20_CeO_0.80_O_2-x_	750	4	46	100	1.655	120,000	Radfarnia et al. (2026)
10Cu-5Fe/CeO_2_	750	1	47	100	0.045	2,400	Chen et al. (2021)
10Fe/CeO_2_	600	1	29	100	2.782	80,000	Watanabe et al. (2023)
15Fe_2_O_3_/5CeO_2_-Al_2_O_3_	600	6	65	87	0.230	15,000	Souza et al. (2026)
5Fe-CeO_2_	700	1	37	100	0.786	18,600	Khandla et al. (2026)
7.5Fe-CeO_2_	600	3	56	100	2.686	60,000	Yu et al. (2025)
20Fe-CeO_2_	500	4	51	100	0.443	60,000	Xie et al. (2026)
20% LHS	750	4	74	82	0.272	12,000	This work
20% HHS	750	4	81	100	0.300	12,000	This work
20% LHL	750	4	85	100	0.315	12,000	This work
20% HHL	750	4	87	100	0.320	12,000	This work
40% HHS	750	4	85	100	0.314	12,000	This work

### 3.3 Post-reaction characterisation of catalyst

Figure 6 displays the XRD patterns for post RWGS reaction samples. The peaks labelled by red dots are identified as centred cubic α-Fe (JCPDS 65-4899) (Batista et al., 2015; Pastor-Pérez et al., 2017). The peak located at 26.3° should be attributed to typical (002) facets of graphite carbon (JCPDS 41-1487) (Liu et al., 2016; Jurkiewicz et al., 2018). Importantly, no new iron oxide peaks were detected, indicating that no bulk oxidization happened during the RWGS process.

**FIGURE 6:**
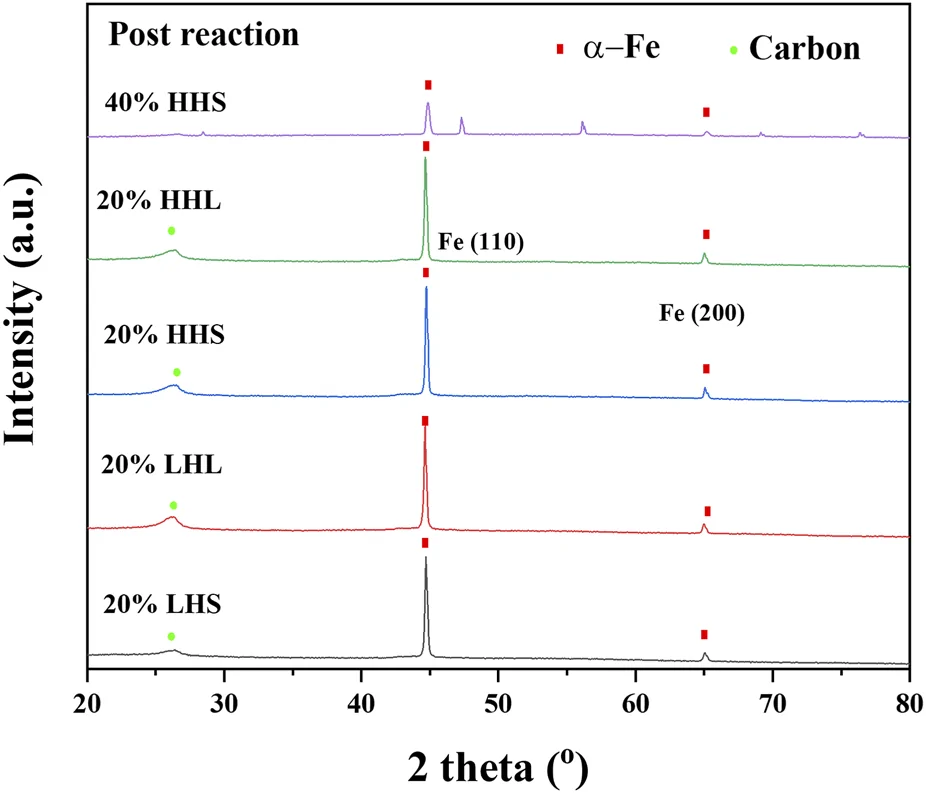
X-ray diffraction patterns for post reaction samples.

Compared to the XRD patterns of fresh catalysts (Figure 1), all the iron oxide and iron carbide peaks disappeared. This is because both H_2_ and CO can be as act as reductant agents for iron oxide and carbide. Previous research confirmed that Fe_3_O_4_ could be reduced to metallic Fe at 615 °C under the atmosphere of 20% H_2_ in N_2_ (Park et al., 2001; Sun et al., 2020). This reduction to metallic Fe can also be confirmed by the change in CO_2_ conversion performance at 550 °C, suggesting that metallic Fe has better catalytic performance compared to Fe_3_O_4_. Similarly, Fe_3_C could be reduced to metallic Fe in CO and H_2_ at 750 °C (Park et al., 2001). In our reactant gas, 40% H_2_ was flowed through the reactor and the temperature reached at 750 °C during the RWGS reaction. Therefore, we may conclude that all the iron carbide and oxide in the fresh samples are reduced to Fe^0^ during the RWGS process. However, since Fe^0^ is the main active phase for the RWGS reaction, the phase transition beneficial to the reaction. Once formed under the reductive reaction environment, metallic Fe remains the dominant crystalline phase after the RWGS reaction, consistent with its proposed role as the main active phase.

## 4 Conclusion

A new family of biochar-based Fe RWGS catalyst display very promising results for the RWGS reaction. Among the developed catalysts, the best-performing samples achieved CO_2_ conversions above 80% and CO selectivities of approximately 100% at 750 °C. The presence of Fe^0^, Fe_3_C and Fe_3_O_4_ all play a role in the catalytic process by showing high affinity towards CO selectivity, with Fe^0^ being the main active phase. The XRD after RWGS showed reduction of Fe_3_O_4_ and Fe_3_C by H_2_ and CO into Fe^0^, while Fe^0^ remained as the dominant active species. Normalization of the CO_2_ consumption and CO formation rates by the experimentally determined Fe content showed that differences in catalytic performance remained after accounting for variations in active-phase loading. Moreover, the catalysts exhibited CO_2_ consumption rates comparable to those reported for other Fe-based RWGS systems.

The key highlight of our findings is that no CH_4_ was detected over the proposed catalysts under the investigated RWGS conditions. Such remarkable result happens even in the low-temperature range where CO_2_ methanation often dominates the selectivity. The Fe/biochar interface, together with the encapsulation and dispersion of the metal particles on the support, may contribute to the observed CO selectivity. All in all, our work serves as a proof-of-concept for the design of eco-friendly catalysts for key green chemistry processes such as the RWGS. Biomass derived supports can indeed play a significant role in CO_2_ conversion scenarios as transition metal carriers offering advantageous structural, thermal and mechanical properties resulting in promising catalytic formulations for real-world applications such as the CAMERE processes or the integrated RWGS-FTS.

## Data Availability

The raw data supporting the conclusions of this article will be made available by the authors, without undue reservation.
